# Evaluation of the 2011 long-lasting, insecticide-treated net distribution for universal coverage in Togo

**DOI:** 10.1186/1475-2875-12-162

**Published:** 2013-05-16

**Authors:** Elizabeth R Stevens, Abigail Aldridge, Yawo Degbey, Akou Pignandi, Monique A Dorkenoo, Justin Hugelen-Padin

**Affiliations:** 1 United States Peace Corps, Togo, BP3194, Lomé, Togo; 2 Ministère de la Santé, Angle Avenue, Sarakawa and Avenue 24 janvier, Lomé, BP336, Togo; 3 Secrétariat Permanent du CCM, Rue des Nations Unies, Lomé, Togo; 4 Faculté Mixte de Médecine et de Pharmacie, Université de Lomé, Lomé, BP1515, Togo

**Keywords:** Malaria, Long-lasting insecticide-treated nets, Universal coverage, Togo

## Abstract

**Background:**

Malaria remains a substantial public health problem in Togo. An integrated child health campaign was conducted in Togo in October 2011. This campaign included a component of free distribution of 2,799,800 long-lasting, insecticide-treated nets (LLINs) to households throughout Togo. This distribution marked the first effort in Togo at universal LLIN coverage and was not targeted specifically to children under five years and pregnant women, but to all household members. This study reports the results of the LLIN distribution campaign in terms of bed net possession and utilization.

**Methods:**

A representative household survey was implemented during the rainy season nine months after the LLIN distribution component of the campaign. Some 6,015 households selected through two stages of probability proportion to size stratified random sampling were interviewed using a brief questionnaire that included a demographic section with questions on the number of household members and sleeping spaces, and a campaign participation section with questions used to evaluate non-LLIN aspects of the campaign. A net roster listed all nets and their characteristics, and a household roster listed all members and visitors with information about bed net use. The questions addressed different aspects of bed net and LLIN possession and utilization. Crude weighted frequencies, percentages, and t- tests of association were calculated using the Stata 12.0 Survey features.

**Results:**

Possession of at least one bed net and/or LLIN increased from 41.3% to 96.7% (P <0.001). Household possession of at least one campaign LLIN was 93.3%. Report LLIN among pregnant women was 77.5% and 79.3% for children under five. For the general population LLIN use was 68.3%.

**Conclusions:**

Due to the gap in LLIN possession and use and the significant number of individuals reporting a lack of nets as a reason for non-use, additional national LLIN distribution campaigns with a stronger educational component need to be implemented in order increase the use of available LLINs and to reach and maintain universal coverage of LLINs in Togo. The LLIN distribution campaign focusing on universal coverage of the general population in Togo was more successful at increasing LLIN possession and use of children under five years and pregnant women than other campaigns focusing only on these target groups.

## Background

Malaria causes an estimated 620,000 to 950,000 infections and over 1,500 deaths per year, predominantly in children under five years old in Togo [1,2]. High levels of insecticide-treated bed net (ITN) use has been shown to reduce malaria mortality and morbidity, and the use of ITNs by the majority of inhabitants can protect vulnerable populations in such communities, including those not sleeping under a mosquito net [3-5].

Togo was the first sub-Saharan African country to adopt the approach of integrated campaigns of child survival interventions to combat the leading causes of childhood mortality. In December 2004, Togo performed the first ever national integrated health campaign, taking advantage of a measles vaccination campaign to distribute insecticide-treated nets, to administer mebendazole tablets and to vaccinate children under five against polio [6-8]. The 2004 distribution of ITNs in combination with vaccinations improved possession rates of ITNs, while minimizing any duplication of delivery costs across the two interventions [9,10]. In December 2008, vitamin A supplementation was coupled with albendazole deworming and the distribution of ITNs to children under five years. During this time, the morbidity rate of malaria measured by external consultations has decreased from 53% in 2007 to 42% in 2008, whereas malaria mortality recorded in hospitals has dropped from 23.1% in 1998 to 20% in 2008 [11].

In October 2011, Togo organized a community-directed national integrated campaign in the context of integrated mass drug administration against three neglected tropical diseases: onchocerciasis, schistosomiasis and soil-transmitted helminthiasis. With the support of partners the campaign included the distribution of long-lasting, insecticide-treated nets (LLINs) for universal coverage, and administration of vitamin A, albendazole, ivermectin and praziquantel. Previous integrated health campaigns distributing ITNs in Togo and other sub-Saharan countries have focused only on vulnerable populations or have not sought universal coverage at national level [6,12-15]. The integrated health campaign in Togo was rolled out in three phases: the census phase, which counted each individual living in a household. The census phase was paired with albendazole deworming and vitamin A supplementation to children under five, and ivermectin distribution, 1–22 August, 2011; the distribution phase of vouchers for LLINs and the distribution of praziquantel, 21–25 September, 2011; the distribution phase of LLINs to all eligible households, 28 September to 4 October, 2011. Campaign LLINs were distributed with the calculation of 1 LLIN for every 2 people in a household, and without regard for previous bed net possession.

To ensure a community-directed approach, the Ministry of Health Plan Togo and Health and Development International (HDI) coordinated the distribution of LLINs with the assistance of community health workers (CHWs). LLINs were distributed by CHWs to voucher holders at distribution points ranging from local health centres, schools, markets, and local and international NGOs [16]. The LLIN distribution component aimed to achieve universal LLIN coverage, defined as one LLIN for every 1.8 people or 0.56 LLINs per person, and addressed the national target goals of increasing bed net usage of pregnant women to 71.4% and 75.4% for children under five [17].

The administrative results obtained after the LLIN distribution showed campaign coverage and LLIN distribution to 99.1% of eligible households. It is important to note that, while the campaign occurred in nearly the entire country, a small densely populated area in the south of Togo including the Golfe district in the Maritime region and the Lomé Commune (20.9% of the population) were not part of the initial LLIN distribution due to lack of available LLINs at the time. A second subsequent distribution of LLINs was implemented in these regions after this study took place and is therefore not included in this evaluation.

In addition to LLINs from the 2011campaign, individuals in Togo have access to nets distributed in past campaigns, net distributed to pregnant women at the time of prenatal check-ups, and those distributed to children under five at their local health centers, and nets sold in the markets. The consistency of mosquito nets available at these sources varies dramatically throughout the country and throughout time. The type of nets, whether ITN, LLIN, or non-impregnated, also varies depending on source and availability at the time. LLINs have a lifespan of only about five years and older ITNs that are not reimpregnated lose their efficacy relatively quickly. For these reasons it has been suggested that LLIN distributions occur approximately every three years.

We report here the results of a household survey to evaluate the LLIN distribution component of the integrated child health campaign in Togo.

## Methods

A cross-sectional household survey was conducted from 3–24 July, 2012, approximately nine months after the LLIN distribution component of the integrated campaign during the rainy season when malaria transmission is at its highest [18]. This investigation took place during the rainy season throughout 29 health districts of Togo where the campaign was implemented. This included all five political regions of Savanes, Kara, Centrale, Plateaux, and Maritime (excluding the Golfe district and Lomé Commune).

### Study design

A two-tiered stratified random sample was used. The primary sampling unit (PSU) was the district (e.g., village) as defined by the 2008 Census adjusted for population inflation, and was chosen randomly by prefecture using an allocation proportional to the population size of the prefecture (PPS). A STEPS sampling spreadsheet provided by the World Health Organization (WHO) was used to select the 193 PSUs using PPS sampling. Stratification by the 29 prefectures involved in the campaign assured the inclusion of ethnic groups, which tend to vary by prefecture. The random sampling of households could not be selected via the STEPS sampling method, due to lack of household addresses, but was conducted by random positioning of surveyors and a random walk method was used to select 20 households within PSUs calculated to achieve a p-value of 0.05.

The survey itself was comprised of 18 close-ended questions in French. The questionnaire included a demographic section with questions on the number of household members and sleeping spaces, and a campaign participation section with questions used to evaluate non-LLIN aspects of the campaign. A net roster listed all nets and their characteristics, and a household roster listed all members and visitors with information about bed net use. Peace Corps Togo was responsible for survey creation and implementation. Oral consent was obtained from survey participants.

### Statistical analysis

Analysis was performed using Stata 12.0 using the Survey features, which account for cluster sampling and unequal selection probabilities. Analyses were weighted, and weights equaled the inverse of the exact probability of selection. Crude weighted frequencies, percentages, and t- tests of association were calculated using the STATA SVY function. Percentages reported in this report reflect this weighting unless otherwise noted. In this report “bed net” refers to any type of bed net while LLIN represents only the LLIN distributed by the integrated campaign of 2011.

### Ethical considerations

The protocol was reviewed and approved by the Togolese Ministry of Health ethics committee. Oral informed consent was obtained from each household representative and individual participants. Exclusion criteria included surveying children under the age of 15. The survey did not collect any sensitive information, involved no invasive procedures, and conferred no significant risk or benefit to participants.

## Results

### Characteristics of households and household members

Of 6,090 selected households, 6,015 (98.8%) were included in the analysis of the survey. Reason for exclusion was “refused to participate.” Of the 6,015 households surveyed, 3,618 (57.9%) had at least one child under five years, 5,093 (83.6%) had at least one woman 15 to 49 years old, and 424 (6.3%) had at least one pregnant woman 15 to 49 years old living in the household. The mean number of individuals per household was 5.8 (Table 1).

**Table 1:** Characteristics of households surveyed by region

**Region**	**Mean number individuals per household (%) [95% CI]**	**Households with at least one child under five years (%) [95% CI]**	**Households with at least one pregnant woman (%) [95% CI]**
Maritime	4.69 [4.43-4.95]	47.98 [45.20-50.77]	4.52 [3.02-6.012]
Plateaux	5.51 [5.11-5.93]	55.28 [51.24-59.32]	3.75 [2.20-5.29]
Centrale	6.38 [6.09-7.02]	64.13 [59.74-68.52]	8.61 [5.24-11.97]
Kara	6.55 [5.86-6.91]	58.34 [53.67-63.03]	8.89 [6.60-11.18]
Savanes	7.29 [6.80-7.79]	75.77 [72.00-79.55]	10.11 [6.99-13.24]
National Total	5.82 [5.63-6.01]	57.89 [56.11-59.66]	6.28 [5.35-7.21]

Almost all of the surveyed households were in rural areas (5,475, 91.0%). A total of 6,360 children under five slept in the surveyed households the night prior to the interview. About one-half (2,312, 49.98%) of these children were female. Altogether, 7,974 women of age 15 to 49 years slept in the households the night prior to the interview, and 441 (5.4%) of these women were pregnant.

### Campaign intervention coverage

Nearly all [5,778, 96.9% (95% confidence interval (CI): 96.1–97.5%)] household respondents interviewed were aware of the LLIN distribution campaign. Of the household respondents aware of the campaign, they learned about it through local town criers (2,893, 54.6%), community health workers (3,253, 52.5%), radio (1,401, 23.3%), local health centre (785, 13.8%), neighbours (450, 8.3%), and television (212, 3.6%).

Most [5,616, 93.4% (95% CI: 92.3–94.6%)] households reported receiving a visit from a CHW as part of the campaign. Of the 5,616 households who received a CHW, 89.7% (4,945) stated that the CHW counted the members of the household, 86.9% (4,843) gave a coupon for the bed net distribution, and 8.6% (519) other, with education (114, 22.0%) making up the largest portion of “other” responses. Many households reported learning how to hang a bed net (5,232, 86.5%) and why sleeping under an LLIN is important (5,480, 90.9%) during the campaign.

Some 98.6% (5,909) of households correctly identified the purpose of sleeping under a bed net; 97.8% (5,872) correctly identified the cause of malaria, however, among households surveyed other responses (876, 14.5%) were provided, including the sun (414, 46.2%), working in the field (201, 22.5%), and “other” (244, 27.3%). The majority of households surveyed rated their appreciation of the campaign as “good” (4,480, 73.5%), the other households rated the campaign as “excellent” (899, 15.3%), “bad” (429, 7.9%), and “no opinion” (202, 3.3%).

### Household LLIN possession

Of households surveyed, 96.7% (5,816) households reported possessing at least one bed net. Nearly all of surveyed households (5,580, 93.3%) had received at least one LLIN from the campaign. At the time of the survey most households (4896, 88.7%) reporting having received at least one campaign net still had at least one campaign LLIN in their possession. Slightly less than half (2,431, 41.3%) of households had at least one non-campaign net representing approximately one-eighth (12.7%) of total nets reported in households. Following the bed net distribution campaign, household possession of at least one bed net rose significantly from 41.3% (2,431) to 96.7% (5,816) (p < 0.001). On average there were 0.45 campaign LLINs per person and 0.57 total bed nets per person. Households with at least one non-campaign net showed a significantly higher net-per-person ratio (0.70) than households possessing only campaign nets resulting from the methods of campaign net distribution, which did not account for previous net possession. The mean number of campaign LLINs per sleeping space was 0.86 and 1.09 for total bed nets, including non-campaign nets, per sleeping space. The mean number of LLINs received per household was 2.4.

### Bed net use

Altogether 18,805 bed nets were identified in the 6,015 households surveyed, of which the survey team visually inspected 78.2% (14,713). About three-quarters (10,635, 74.0%) of all inspected bed nets were hung for use. Of the 35,419 people reporting sleeping in their households the previous night, fewer than three-quarters [25,896, 71.4% (95% CI: 69.3–73.5%)] reported sleeping under a bed net the previous night. Of the 9,523 people reporting not sleeping under a bed net the previous night, the most common reasons for not using a bed net were not receiving a net or receiving insufficient nets from the campaign (861, 21.24%), too hot under a net (219, 7.8%), no mosquitoes at the time (168, 5.7%), and “other” (1,006, 34.5%) which included bed net not hung (125, 12.8%), bed net being washed (144, 12.8%), being “physically incapable,” “lacking the materials necessary,” and “lacking the knowledge” to hang the bed nets (117,11.9%), and lack of space (97, 9.1%).

The most common sources of bed nets reported by households with at least one bed net were a campaign (4,482, 95.2%), services from a health centre (1,032, 22.8%), and purchase (549, 12.8%). In younger age groups, including children under five and between the age of 6 and 14, there was no significant difference between the sexes as to who slept under a bed net the previous night. In the older age groups however, a significant difference can be seen between the sexes with a higher percentage (75.5%) of women age 15–49 sleeping beneath a bed net than men (69.4%) and in people 50+ more men (73.9%) sleep under a bed net than women (66.0%) (p < 0.001) (Table 2).

**Table 2:** Percentage bed net usage by age and sex

**Age group**	**Individuals sleeping under bed net previous night (%) [95% CI]**	**Males sleeping under bed net previous night (%) [95% CI]**	**Females sleeping under bed net previous night (%) [95% CI]**	**P-value**
Age <5	81.5 [79.4-83.6]	81.3 [78.4-84.1]	81.3 [79.1-83.6]	0.34
Age 5-14	70.1 [67.7-72.5]	70.2 [67.7-72.7]	69.8 [67.2-72.5]	0.15
Age 15-49	71.8 [69.6-74.0]	69.4 [67.0-71.7]	75.5 [73.2-77.8]	<0.001
Age 50+	69.1 [66.6-71.7]	73.9 [71.2-76.8]	66.0 [62.9-69.3]	<0.001

### Bed net use by children under five years

Use of bed nets by children under five years was considerably higher than the mean of the general population sleeping beneath a bed net the previous night. Among children under five, the majority [79.3 (95% CI: 76.8-81.80] were reported sleeping under an LLIN the previous night while 81.5% (95% CI: 79.4–83.6%) reportedly slept under a bed net of any time including LLINs.

### Bed net use by pregnant women

As with children under five, the proportion of individuals sleeping beneath a bed net the previous night is higher for pregnant women than for the general population. Of the 427 pregnant women who slept in the household the previous night 77.5% slept under a LLIN, while 80.9% (347) reported sleeping under any type of bed net including LLINs.

### Regional differences

Togo is divided into six sanitary regions and the national averages do not always represent the reality in each region for every indicator. Maritime (1,036/1,240, 89.0%) and Plateaux (1,120/1,335, 91.9%) reported fewer households reporting receiving at least one campaign bed net than the mean (5,580/6,015, 93.3%). Maritime (1,827/2,733, 67.7%) and Kara (1,720/2,543, 71.5%) showed a lower proportion of bed nets being hung than the mean (10,395/14,713 74.1%), while Plateaux (2,308/3,151, 76.3%), Centrale (2,200/2,837, 79.0%), and Savanes (2,580/3,449, 78.1%) showed an above average number being hung (Table 3).

**Table 3:** Household LLIN and bed net possession by region

**Region**	**Households w/at least one bed net(%) [95% CI]**	**Households receiving at least one LLIN from campaign(%) [95% CI]**	**Households possessing at least one non-campaign net(%) [95% CI]**	**Mean individuals stated living per household(#) [95% CI]**	**Mean LLINs received per household(#) [95% CI]**	**Mean campaign LLINs per person(#) [95% CI]**	**Mean campaign LLINs per sleeping space(#) [95% CI]**
Maritime	94.76 [93.13-96.39]	89.03 [86.43-91.63]	39.19 [35.05-43.34]	4.69 [4.43-4.95]	1.88 [1.79-1.98]	.46 [.44-.49]	.82 [.78-.86]
Plateaux	96.10 [94.22-97.99]	91.91 [88.94-94.88]	46.22 [41.27-51.17]	5.52 [5.11-5.93]	2.21 [2.06-2.36]	.45 [.42-.47]	.84 [.79-.89]
Centrale	96.65 [94.86-98.43]	94.11 [92.03-96.20]	25.54 [20.34-30.75]	6.56 [6.09-7.02]	2.70 [2.52-2.88]	.44 [.42-.46]	.91 [.85-.97]
Kara	97.63 [96.41-98.86]	95.00 [93.20-96.80]	39.84 [33.97-45.71]	6.38 [5.86-6.91]	2.78 [2.57-2.99]	.48 [.45-.51]	.93 [.87-.997]
Savanes	98.45 [97.61-99.29]	94.21 [91.14-97.27]	49.10 [42.65-55.56]	7.30 [6.80-7.79]	3.01 [2.76-3.27]	.43 [.40-.46]	.86 [.80-.92]
National Total	96.40 [95.63-97.18]	92.24 [90.97-93.50]	41.32 [38.97-43.66]	5.82 [5.63-6.01]	2.39 [ 2.31-2.47]	.45 [.44-.47]	.86 [.84-.88]

Bed net usage was lowest among Maritime: only 63.8% (3,686/5,704) of people who reported having slept in their household the previous night reported sleeping beneath a bed net and only 58.9% beneath a LLIN. In the region of Maritime fewer children under five were reported sleeping under a LLIN (71.8%) than the mean (79.3%). The number of pregnant women reported sleeping under a LLIN was also lower in Maritime (63.9%) than the mean (77.5%) in the other regions (Table 4).

**Table 4:** Household bed net and LLIN use by region

**Region**	**Observed bed nets hung for use (%) [95% CI]**	**Individuals reporting sleeping under a bed net the previous night(%) [95% CI]**	**Individuals reporting sleeping under a LLIN the previous night(%) [95% CI]**	**Children <5 years reported sleeping under a bed net the previous night (%) [95% CI]**	**Children <5 years reported sleeping under a LLIN the previous night(%) [95% CI]**	**Pregnant women reporting sleeping under a bed net the previous nightNB: missing standard error because of multiple stratum with single sampling unit(%) [95% CI]**	**Pregnant women reporting sleeping under a LLIN the previous nightNB: missing standard error because of multiple stratum with single sampling unit(%) [95% CI]**
Maritime	68.55 [62.39-74.72]	63.85 [58.75-68.93]	58.92 [53.45-64.39]	75.85 [70.46-81.25]	71.83 [65.31-78.35]	69.64	63.89
Plateaux	76.28 [72.62-79.94]	73.34 [69.49-77.19]	68.17 [63.71-72.63]	82.88 [78.58-87.19]	79.89 [74.63-85.16]	86.00	83.33
Centrale	78.99 [74.79-83.18]	74.11 [69.74-78.47]	72.49 [67.44-77.54]	83.91 [79.33-88.49]	82.52 [77.45-87.60]	82.11	79.41
Kara	71.49 [65.81-77.16]	71.18 [66.03- 76.33]	70.30 [64.82-75.77]	79.67 [74.68-84.66]	77.88 [72.04-83.72]	80.75	80.03
Savanes	78.06 [72.61-83.52]	78.97 [74.80-83.13]	78.42 [73.43-83.40]	85.81 [81.98-89.64]	85.56 [80.61-90.52]	85.35	80.95
National Total	71.49 [65.81-77.16]	71.39 [69.29-73.49]	68.23 [65.88-70.58]	81.70 [79.60-83.81]	79.31 [76.80-81.81]	80.91	77.49

## Discussion

The campaign for the distribution of LLINs successfully reached the population, with vast majority of households being aware of the campaign, receiving a visit from a CHW, and receiving at least one LLIN from the campaign. Although the campaign failed to attain its goal of universal bed net coverage of at least one LLIN for every 1.8 people, it rapidly increased household LLIN possession in all households including those left out of previous campaigns that focused on children under five and pregnant women.

While knowledge acquisition cannot be confirmed as directly related to campaign activities, malaria awareness has been generally achieved, as nearly all households correctly identified the purpose of sleeping under a mosquito net and correctly identified the cause of malaria, though 14.5% also provided an incorrect response in addition to the correct one. While the vast majority of households reported learning how to hang a mosquito net as a result of the campaign, and gained understanding why sleeping under a LLIN is important. Although the majority of households were able to demonstrate knowledge of malaria and LLINs, the roughly 17% of people that provided at least one incorrect response demonstrate the continued need for malaria awareness efforts.

Based on bed net possession, bed net usage was lower than expected. Although the campaign successfully reached the population, less than three-quarters of LLINs were found to be hung and a similar percentage of people reported sleeping under a bed net. The region of Maritime showed exceptionally low usage, with only two-thirds of campaign nets being hung and even fewer people reporting sleeping under a bed net. Maritime also reported the lowest number of households that received a visit from a CHW, and the fewest households understanding how to hang a bed net and why sleeping under a bed net is important. With nearly a third of already distributed nets not being used, informational campaigns to increase bed net use can strengthen the efficiency of the mosquito net distribution so as to reduce malaria morbidity [12].

While the most common reason for not sleeping under a net was insufficient nets in the household, the second most common reason (11.9%) for not sleeping under a bed net the previous night related to a bed net not being hung. Households reported being “physically incapable,” “lacking the materials necessary,” and “lacking the knowledge” to hang the bed nets received during the campaign. By increasing efforts to assist people to attain the materials, manpower and knowledge of how and why to hang a bed net, the 25% of distributed LLINs not currently hung can be put into use, increasing effective bed net usage with nets that have already been distributed and ultimately reinforcing the efficacy of universal LLIN coverage.

Achieving universal bed net coverage as defined in this campaign by one net for every 1.8 people, may not be sufficient to protect and cover all of the Togolese population. While 90% of universal bed net coverage was achieved, the mean number of bed nets per sleeping space was only 0.86 indicating that in reality there exist more sleeping spaces than assumed by the current definition of universal coverage. While universal coverage will successfully cover much of the population, a number of individuals will be left uncovered by the use of the standard of two people per sleeping space, as not all household members share sleeping spaces. Future campaigns may consider taking into account the number of sleeping spaces in addition to number of household members in order to avoid leaving a portion of the population uncovered.

In addition to the general goal of universal coverage, the success of the campaign was in line with the objectives for minimum effective coverage levels of the National Health Development Plan (NHDP) 2012–2015: 71.4% of pregnant women and 75.4% of children under five sleeping under a LLIN [17]. From the 2010 baseline of pregnant women and children under five sleeping under an ITN, the 2011 campaign successfully increased ITN use by these target groups [17]. By nearly 10 percentage points for pregnant women and five percentage points for children nationally, the 2011 bed net campaign has successfully achieved the goals set by NPHD for LLIN use. Both goals were, however, not successfully achieved in the region of Maritime, where the number of pregnant women sleeping beneath a LLIN remains at 63.9%. The bed net usage of children under five in Maritime reaches the NHDP goal, but not for LLIN use specifically, and is the lowest bed net usage for children under five, compared to the mean of all five regions.

As the issues surrounding the logistics of bed net distribution are addressed, so that bed net availability no longer hinders bed net usage, social and culture factors may need to be dealt with next to increase bed net usage and decrease disparities. The differences that are apparent in bed net usage between genders and age groups may point to cultural factors in sleeping arrangements and household politics that affect bed net utilization by household members. With children showing a significant drop off in bed net usage after the age of five, efforts to identify the factors behind this decrease are important in order to delineate interventions that will potentially lead to long-term behavior change and continued bed net usage through adulthood.

While bed net usage was not 100%, the integrated campaign distributing LLINs to all people regardless of age, with the purpose of achieving universal coverage, was more successful at covering vulnerable populations than previous campaigns performed in Togo and other sub-Saharan countries focusing solely on target groups such as pregnant women and children under five [7,14,19-23]. The universal coverage campaign also successfully covered other groups at high risk to malaria infection, such as school-aged children that have been shown to have low bed net usage rates and are not covered by other types of ITN distribution campaigns [24-30].

This study had several limitations. The cross-sectional design did not allow for the understanding of how bed net use and possession might vary over time and according to seasonal fluctuations. Due to the wide variety of sources and a lack of brand labels on nets not distributed by this campaign the information collected about the non-campaign nets was limited and unable to confirm the type of net thus limiting the specificity of the analysis of total LLIN and ITN use. Moreover, only self-reported use of bed nets by household members was ascertained, and beyond visual inspection of a portion of the nets there was no means to confirm data, such as the number of nets in use, the number of pregnant women and the number of people who stayed in the household and under a bed net the night before the interview. It is common in Togo that a woman does not confirm her pregnancy until the final months that she is pregnant, potentially underestimating the number of pregnant women in the surveyed population. While efforts were made to randomize selection of households, there exists the potential of selection bias as selection was made by the surveyors and not from a simple random sample.

## Conclusions

In conclusion, the LLIN distribution component of the 2011 integrated campaign was effective in rapidly increasing household possession and use of bed nets, achieving national bed net coverage goals set by NHDP 2012–2015. Given the significant rates of non-usage of distributed bed nets, future campaigns should include further informational and capacity building components. The campaign did not fully achieve its goal of universal coverage, highlighting the need for further efforts including future national LLIN distribution and education campaigns to increase LLIN possession and bed net use. While not achieving full universal coverage, the integrated campaign distributing LLINs to the entire population was more successful at increasing bed net possession rates of vulnerable populations and the population in general than campaigns directed towards target groups alone.

## Abbreviations

CHW: Community health worker; HDI: Ministry of Health Plan Togo and Health and Development International; ITN: Insecticide-treated net; LLIN: Long-lasting, insecticide-treated net; NHDP: National Health Development Plan; PPS: Population size of the prefecture; PSU: Primary sampling unit.

## Competing interests

The authors declare that they have no competing interests.

## Authors  contributions

ERS contributed to the study design, conducted the research, performed statistical analysis, collected and interpreted the data, and drafted the manuscript. AA collected and interpreted data, conducted the research, and helped draft the manuscript. YD and AP participated in the design of the study. MAD helped draft the manuscript. JHP obtained funding for the project, conceived the study, contributed to the study design, collected data, designed data collection software, and conducted the research. All authors contributed to the study design. All authors read and approved the final manuscript.
